# Estimating the incidence of heart failure: Insights from an illness-death model using statutory health insurance data from 70 million people in Germany

**DOI:** 10.1371/journal.pone.0341810

**Published:** 2026-02-02

**Authors:** Mike Wagner, Dennis Rottländer, Sabrina Voß, Ralph Brinks

**Affiliations:** 1 Chair of Medical Biometry and Epidemiology, Witten/Herdecke University, Witten, Germany; 2 Department of Cardiology, Krankenhaus Porz am Rhein, Cologne, Germany; 3 Department of Cardiology, Witten/Herdecke University, Witten, Germany; PRISM CRO, PAKISTAN

## Abstract

**Introduction:**

Heart Failure (HF) is a considerable public health issue that affects more than 64 million people worldwide, with a prevalence of 1% to 3% in Western industrialized countries. However, reliable data on the incidence of HF are lacking, making it difficult to assess. The aim of our study was to estimate the age- and sex-specific incidence of HF in Germany using the illness-death model (IDM) and an associated partial differential equation (PDE), based on aggregated prevalence and mortality data.

**Methods:**

Prevalence data for 2009–2017 were used from the Central Institute for Statutory Health Insurance (Zi). Data on HF mortality were taken from a comparable Norwegian population from 2013. A PDE using a bootstrapping approach was used to calculate HF incidence. The estimated incidence was the median of 5,000 samples, complemented by 95% bootstrapping confidence intervals (CIs). We ran a ± 15% calibration sensitivity analysis by uniformly scaling Norwegian Mortality rate ratios (MRRs) to test transferability to the German population. The incidence was then re-estimated with the full 5,000-replicate bootstrap. Medians with 95% empirical intervals were reported for each scenario.

**Results:**

In all age groups, men had a higher incidence than women. The highest incidences were estimated in age groups over 90 years old, with approximately 88/1,000 person-years (py) (95% CI: 73–102) for men and 45/1,000 py (95% CI: 29–61) for women. A sharp increase in incidence rates could be seen in men from the age of 60 years and in women from the age of 65 years. The slope of the incidence was less pronounced among women aged above 60 years compared to men, without impacting the overall trend of increased incidence in higher age groups for women. Uniform ±15% scaling shifted incidence levels, but preserved the age-specific shape and male–female ordering. The effects were small to moderate in age groups from 50 to 90, and negligible in age groups ≤45, with the largest absolute shifts observed at very old ages.

**Conclusion:**

The IDM and an associated PDE were used to estimate a rising incidence of HF with age for both men and women. These findings underscore the importance of age- and sex-specific approaches in HF prevention and management strategies, particularly in older populations where the burden of HF is most pronounced. The method complements existing estimation techniques while circumventing the necessity of costly follow-up studies.

## Introduction

Since the turn of the century, cardiovascular diseases, including chronic heart failure (HF), have been described as an emerging global epidemic [1,2]. Today, HF remains an enormous public health issue, affecting more than 64 million people worldwide, particularly in higher age groups (> 65 years). With a prevalence of 1% to 3% in Western industrialized countries, it is an important factor in morbidity, mortality, and quality of life [3]. While the incidence of HF has stabilized and appears to be declining in industrialized countries, the prevalence is increasing due to aging populations, improved treatment and survival rates for ischemic heart disease, and the availability of effective evidence-based therapies [3–5]. Notably, the opposite trend has been observed in people younger than 50 years in several countries in the last decades [6–8]. This may be related to the increase in obesity and unhealthy lifestyle factors. In line with global and regional trends, the high prevalence and adverse profile of hyperglycemia and dyslipidemia reported by Sarfraz et al. [9] underscore the importance of cardiometabolic risk factors for interpreting the contemporary burden of HF. Despite the immediate relevance of the disease, data on prevalence and especially incidence are inconsistent or insufficient in the German population [10–13]. Incidence is considered an important epidemiological parameter for the development of a disease, providing information on the need for preventive measures or other interventions. It is therefore imperative to be able to make accurate and readily available statements about HF incidence. One important reason for the limited availability of incidence data is the high costs and long duration associated with conducting longitudinal cohort studies. Furthermore, projections of future prevalence and estimates of the number of patients with HF are subject to a high degree of uncertainty, as they require a valid estimate of incidence. For example, the disease-free intervals method, used in recent publications [11,13], carries a high risk of overestimating the incidence [14].

We aimed to estimate the incidence of HF in Germany for the period from 2009 to 2017 using a partial differential equation (PDE) [15] based on an illness-death model (IDM), and compared the results with published incidence data. Both PDE and IDM have been successfully used to predict future prevalence and estimate incidence for other diseases, such as diabetes mellitus or Parkinson’s disease [16–19]. Using a PDE for incidence estimation does not require longitudinal cohort studies or disease-free intervals, so an overestimation of incidence is less likely and there is a lower potential of bias.

## Methods

### Illness-death model and partial differential equation

**Fig 1:**
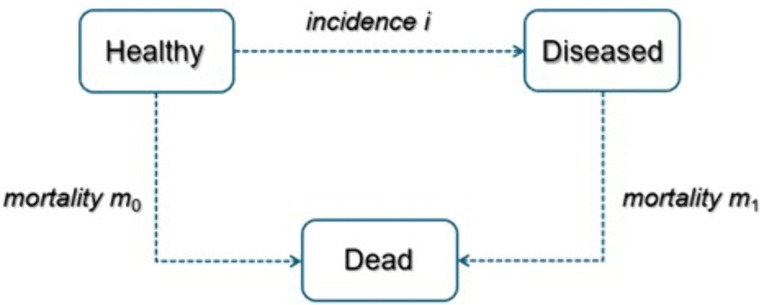
Illness-death model. The illness-death model with mortality and incidence rates as transition intensities between states.

Fig 1 shows an IDM without recovery, a multistate model first described by Keiding in 1991 [20]. This model displays a chronic condition in a population with three states, “Healthy”, “Diseased”, and “Dead”, using the incidence rate i (Healthy → Diseased), the mortality rate m_0_ of healthy (Healthy → Dead), and the mortality rate m_1_ of diseased (Diseased → Dead) as transition intensities between the states. The transition rates depend on two time-scales: age in years (a) and calendar time (t) (i(t,a), m_0_(t,a), and m_1_ (t,a)). Therefore, the IDM describes the dynamics and relationships of prevalence, incidence, and mortality for a chronic disease.

A PDE can be used to quantify the relationship between transition rates and the prevalence of the chronic condition of interest by describing the changes in prevalence (p), depending on the rates in the IDM (i, m_0_, m_1_) [15]:


$$\left(\frac{\partial }{\partial t}+\frac{\partial }{\partial a}\right)p=(\text{1}-p)\left[i-p\left(m_{\text{1}}-m_{\text{0}}\right)\right]$$

Using the general mortality $m=pm_{\text{1}}+(\text{1}-p)m_{\text{0}}\;$ and the mortality rate ratio $MRR=\frac{m_{\text{1}}}{m_{\text{0}}}$, the PDE can be solved for the incidence:


$$i=\frac{\left(\frac{\partial }{\partial t}+\frac{\partial }{\partial a}\right)p}{\text{1}-p}+m\frac{p(MRR-\text{1})}{\text{1}+p(MRR-\text{1})}.$$ (A)

Formula (A) can therefore be used to calculate the incidence rate of a chronic disease. In this work, HF is assumed to be a disease without recovery (no transitions back from *diseased* to *healthy* state), so the transitions in the chosen model are irreversible.

### Data

**Fig 2:**
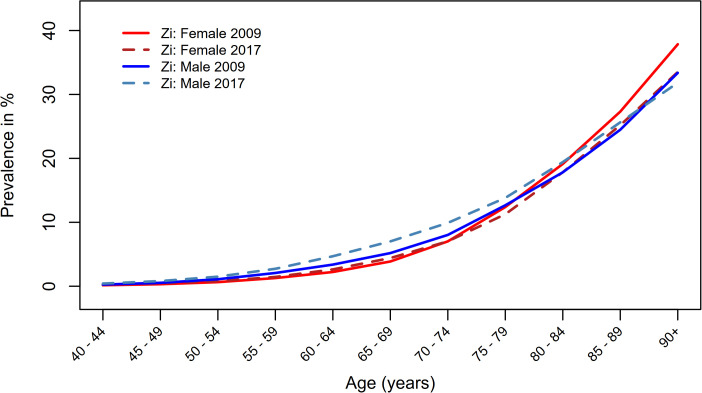
Prevalence of HF. HF prevalence in Germany for 2009 and 2017, published by the Zi [12], stratified by age and sex.

Our analysis is based on prevalence data published in 2018 by the Central Institute for Statutory Health Insurance (Zi) in the ‘Care Atlas’ (Versorgungsatlas, Report No. 18/09) [12]. The survey covered the period from 2009 to 2017. This database included all patients with statutory health insurance and was derived from accounting data for services provided by contract physicians at least once in the respective year. This included a total population of 69,719,142 people with statutory health insurance in 2009 and 71,809,505 insured persons in 2017. The aggregated data used can be considered representative with a low selection bias, as it covers more than 85% of the German population. However, approximately 12.5% of the German population is privately insured and not captured in Zi claims; this may introduce selection bias due to systematic differences in health status and care pathways between private and statutorily insured individuals [23,24]. Data records were anonymized, and also included sociodemographic characteristics, comorbidities, billed services, and medical history documented by the physicians. Patients were considered prevalent cases if they had a diagnosis of HF in at least two quarters within 12 months (M2Q-Methodology) to exclude patients with only a suspected diagnosis of HF. Classification was performed according to the diagnosis code ICD-10-GM (International Statistical Classification of Diseases, 10th revision, German modification) with the ICD-10 codes I50, I11.0, I13.0, and I13.2; these contain the main diagnosis of HF as well as other conditions associated with the development of HF. We used the same ICD codes in our analysis, as well as aggregated sex- and age-specific prevalence data on HF, as published by Holstiege et al. [12]. There was no need for approval by an ethics committee, as only anonymized and aggregated data were used for the analysis [12,22]. The Zi reported the prevalence of HF stratified by age, sex, and region for the statutorily insured population as diagnostic-prevalence in percentage for the years 2009–2017 (see Fig 2). Given the low prevalence of HF in individuals under 20 years, our analysis focused on prevalent cases among men and women aged 20–109 years. Of the 69,719,142 individuals recorded in 2009, a total of 2,044,921 people were diagnosed with HF, of whom 1,239,472 (60.6%) were women and 805,499 (39.4%) were men. For 2017, the number of prevalent HF cases had risen to 2,459,995 from 71,809,505 recorded individuals, comprising 1,330,358 (54.1%) women and 1,129,637 (45.9%) men.

Formula (A) was used to calculate incidence. HF-prevalence data (for 2009 and 2017) and the mortality of the population were needed, including the mortality rate ratio (MRR) of persons with HF and without HF. Prevalence data for 2009 and 2017 were extracted from the Zi-dataset. Data on general mortality in the German population were taken from “The Human Mortality Database” (HMD), most recently recorded in 2020 [21]. HMD-datasets were downloaded on October 12, 2021, and therefore correspond to the latest survey status at the time of processing.

Because age-specific German estimates of MRRs for heart failure were unavailable, we identified external MRR values through a systematic literature search in MEDLINE (see S1 Text). This resulted in the use of age- and sex-specific MRRs from a Norwegian study reported by Ødegaard et al. [22]. In the study, MRRs were derived to the contemporaneous Norwegian general population in 5-year age-by-sex strata, with expected deaths calculated by applying contemporaneous Norwegian all-cause mortality rates (reported by Statistics Norway/Statistisk sentralbyrå) to the population at risk in each age-sex stratum (MRR = observed deaths/ expected deaths). To reflect this definition and provide clarity throughout our study, we consistently use the term “age-specific MRRs” or “MRRs”, rather than the term “SMR” (standardized mortality ratio) used by Ødegaard et al. [22]. For alignment with our prevalence reference, we selected the reported 2013 MRR estimates. To obtain a smooth age function for the PDE-based incidence estimation (see Formula A), we fitted sex-specific log-linear models to the 5-year age-group MRRs from Norway. At each age, these models yield the model-predicted (fitted) MRRs. S1 Fig in the provided Supporting Information displays the observed age-specific MRRs together with the corresponding model-predicted (fitted) MRRs from the sex-specific log-linear fits. The curve appears non-linear on the original scale because a linear relationship on the log scale becomes exponential after back-transformation. The fit was strong (adjusted R² = 0.9271). This regression pertains to the MRR input only. Prevalence was modeled separately on the logit scale using a natural-cubic-spline basis with interactions by time and sex. To assess transferability from Norway to Germany, we additionally performed a ± 15% one-way sensitivity analysis by scaling all age-specific MRRs (see Results – Sensitivity Analysis and Supporting Information S1 and S2 Tables). Evidence that MRRs are shown to be stable across populations further motivated this approach [25].

### Statistical analysis

Statistical analysis was performed using R (The R Foundation for Statistical Computing), version 4.4.2, and the RStudio development environment. The R packages “splines” and “stats” were used to extend computational and graphical capabilities. The source code for the graphical representation of the incidence rates is available on the platform Zenodo (see Data availability statement).

**Fig 3:**
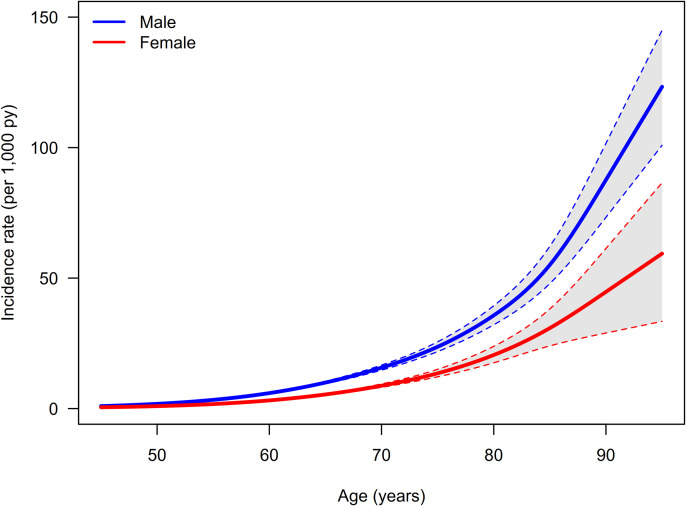
Incidence of heart failure. Median age-specific incidence (solid lines) with 95% bootstrap intervals (shaded grey band and dashed lines) for men (blue) and women (red). Curves are drawn by natural cubic spline interpolation of the bootstrap median and 2.5th/97.5th percentiles at the age-group midpoints. The spline does not contribute uncertainty. Exact point estimates and 95% confidence intervals per age group are reported in Table 1.

For incidence estimation, we used a parametric bootstrap at the model level, appropriate for aggregated summaries. No individual records were resampled or excluded. The unit of analysis was the age-by-sex stratum on an age grid from 20–90 years in 5-year increments, with 2013 as the anchor year between the prevalence years 2009 and 2017. Following Efron and Tibshirani [26], we ran 5,000 iterations. In each iteration, we (i) perturbed the prevalence model’s linear predictor on the logit scale by adding a normal deviate with standard deviation equal to its estimated standard error; (ii) perturbed the sex-specific log-linear MRR regression by adding normal deviates to the intercept and slope according to the estimated standard errors; and (iii) kept general population mortality fixed. We then (iv) recomputed the Lexis-direction derivative and solved the PDE (see Formula A) to obtain age- and sex-specific incidence. Across iterations, point estimates were the medians of the age- and sex-specific incidences. Uncertainty is expressed as 95% empirical bootstrap intervals (2.5th/97.5th percentiles), which quantify variability and precision arising from parameter uncertainty in the prevalence and MRR models. They do not establish accuracy (absence of bias) and do not reflect individual-level sampling variability, model-selection uncertainty, or unmeasured systematic bias. Potential bias from using Norwegian MRRs [22] was examined separately via a ± 15% sensitivity analysis (see Results – Sensitivity analysis). For visualization, we used a natural cubic spline interpolant defined on the age support points (20–90 years in 5-year steps), which serve as the knots. No additional internal knots or smoothing penalties were used. The spline strictly interpolates the bootstrap median, the 2.5th and 97.5th percentile series, and is evaluated on a finer age grid for plotting. It does not add uncertainty or weights. Table 1 reports the bootstrap medians and 95% empirical intervals at the support ages, while Fig 3 shows the interpolated curves with the corresponding 95% interval band.

**Table 1:** Incidence of heart failure in Germany per 1,000 person years. Calculated age-specific heart failure incidence by sex as bootstrap medians (point estimates) with 95% empirical (bootstrap) intervals (2.5th/97.5th) at the support ages (20 to 90 years by 5-year steps). The exact point estimates and 95% confidence intervals (CIs) for each age group are listed, with values rounded to three decimals.

Age (years)	Males	95% CI	Females	95% CI
**20–24**	0.040	[0.039; 0.041]	0.031	[0.030; 0.031]
**25–29**	0.075	[0.074; 0.076]	0.052	[0.051; 0.052]
**30–34**	0.141	[0.139; 0.143]	0.088	[0.087; 0.089]
**35–39**	0.267	[0.263; 0.272]	0.154	[0.151; 0.156]
**40–44**	0.507	[0.497; 0.518]	0.274	[0.269; 0.280]
**45–49**	0.963	[0.941; 0.985]	0.502	[0.491; 0.514]
**50–54**	1.816	[1.771; 1.863]	0.937	[0.915; 0.961]
**55–59**	3.343	[3.246; 3.447]	1.734	[1.686; 1.790]
**60–64**	5.931	[5.714; 6.164]	3.136	[3.022; 3.268]
**65–69**	9.965	[9.499; 10.468]	5.417	[5.149; 5.730]
**70–74**	15.678	[14.737; 16.649]	8.757	[8.152; 9.439]
**75–79**	23.681	[21.841; 25.582]	13.522	[12.173; 15.043]
**80–84**	35.722	[32.113; 39.401]	20.540	[17.543; 23.871]
**85–89**	55.055	[47.911; 62.172]	30.842	[24.026; 38.180]
**90+**	87.638	[73.286; 101.613]	44.735	[28.957; 61.339]

### Sensitivity analysis for MRR calibration

To assess robustness to potential miscalibration of the Norwegian MRRs [22] when applied to the German population, we performed a brief sensitivity analysis. We multiplied all age- and sex-specific Norwegian MRRs [22] by uniform factors of +15% and −15% (in two separate scenarios), in addition to the baseline (unmodified) MRRs. For each scenario, we re-ran the complete estimation, including the bootstrap with 5,000 replicates, and summarized the age-specific incidence by the median, 2.5th, and 97.5th percentiles. In each bootstrap replicate, we (i) perturbed the prevalence predictions via their model-based standard errors, and (ii) perturbed the regression coefficients (intercept and slope) of the sex-specific log-linear models for the Norwegian MRRs [22] by small random offsets scaled to their estimated standard errors. German all-cause mortality was treated as fixed. The same random draws were reused across the three MRR scenarios, enabling matched (paired) comparisons of baseline versus ±15% scaling. This set-up probes robustness to a global multiplicative calibration error while preserving its age-pattern. It does not address possible age- or sex-differential deviations, nor uncertainty in German all-cause mortality. All scenarios described are reported in detail in the Supporting Information (see S1 and S2 Tables).

## Results

### Age- and sex-related incidence rate of heart failure

Incidence rates of HF, stratified by age and sex per 1,000 person-years (py), were estimated based on previously reported prevalence data from the Zi (see Fig 2) and Norwegian mortality data (see Table 1 and Fig 3). The estimated incidence (median of the 5,000 bootstrap samples) was higher in the male population across all age groups. Little variability of incidence rates was observed in age groups younger than 50 years, with only minor differences between men and women. Differences in median incidences increased and differed more sharply in age-groups of 60–64 (Males: 5.931/1,000 py [5.714; 6.164], Females: 3.136/1,000 py [3.022; 3.268]) and above. The increase reached a maximum in both sexes at ages over 90 years. Men had a maximum of approximately 87.638/1,000 py [73.286; 101.613] and women of approximately 44.735/1,000 py [28.957; 61.339]. The slope of the incidence was less pronounced among women in the age group above 60 years compared to men, but this did not negate the overall trend of a continual increase in higher age groups for women. A sharper increase in incidence rates can be seen in men from the age of 60 years and in women from the age of 65 years. The calculated median incidences of HF for men and women across all age groups, along with the 95% confidence intervals (CIs), are provided in Table 1.

### Precision and uncertainty of the incidence estimates

The 95% bootstrap CIs, based on 2.5% and 97.5% quantiles of the estimated incidence in 5,000 random samples, were calculated to analyze precision and uncertainty in the incidence estimation. Fig 3 shows the median age-dependent incidences and the bootstrap confidence intervals as gray areas in the dashed lines. The bootstrap intervals get wider with increasing age, indicating greater uncertainty in the estimates for the older age groups. As an example, at age 90, the 95% CI was 28.957 to 61.339 for women and 73.286 to 101.613 for men (Table 1 and Fig 3).

### Sensitivity analysis

A ± 15% calibration sensitivity analysis of Norwegian MRRs [22] was conducted to evaluate the robustness of incidence estimates when applying these ratios to the German population. Uniform ±15% scaling shifted incidence levels accordingly, while leaving the age-specific shape and qualitative ordering unchanged. For men, median incidence under the + 15% scenario exceeded the baseline by approximately 1.6%–11.2% across ages 50–90 (e.g., 15.68 vs. 16.46 per 1,000 py at age 70 and 87.64 vs. 97.45 per 1,000 py at age 90). Under the −15% scenario, medians were approximately 2.2%–13.1% lower over the same age range (e.g., 14.82 vs. 15.68 per 1,000 py at age 70 and 76.16 vs. 87.64 per 1,000 py at age 90). For women, the + 15% scenario increased medians by approximately 1.1%–16.5% across ages 50–90 (e.g., 8.76 vs. 9.10 per 1,000 py at age 70 and 44.74 vs. 52.14 per 1,000 py at age 90), while the −15% scenario decreased medians by 1.7%–18.7% (e.g., 8.39 vs. 8.76 per 1,000 py at age 70 and 36.38 vs. 44.74 per 1,000 py at age 90). At younger ages (≤45 years), differences for men and women were negligible at the reported precision.

Uncertainty increased with age and was similar across scenarios. For men, the 95% empirical interval width grew from approximately 0.02 per 1,000 py at age 40 to 28.3 per 1,000 py at age 90 in the baseline scenario (73.29–101.61 per 1,000 py at age 90), with comparable widths under +15% (83.16–111.19 per 1,000 py) and −15% (61.93–90.23 per 1,000 py). For women, the corresponding baseline widths increased from around 0.01 per 1,000 py at age 40 to around 32.4 per 1,000 py at age 90 (28.96–61.34 per 1,000 py), again with similar widths under +15% (35.83–68.88 per 1,000 py) and −15% (21.29–52.62 per 1,000 py). The shifts induced by ±15% scaling remained modest relative to this age-related uncertainty. For example, at age 90, the + 15% shift equaled approximately 9.8 per 1,000 py for men and 7.4 per 1,000 py for women, compared with interval widths of around 28.3 and 32.4 per 1,000 py, respectively. More detailed medians and 95% empirical intervals for all three scenarios are provided in the Supplementary Information (see S1 and S2 Tables).

## Discussion

Based on prevalence and mortality data, the incidence of HF for men and women in Germany between 2009 and 2017 was calculated using an IDM for HF as a chronic condition and a corresponding PDE. The main results of our study are as follows:

1. Men had a higher incidence of HF than women across all age groups.
2. The estimated incidence increased quickly and differed between sexes more sharply from age 60 years and above, with a less pronounced slope of incidence for women in the higher age groups.
3. For both sexes, the highest median incidence was observed in age groups over 90 years, with no evidence of a decline at older ages.

The ± 15% calibration sensitivity analysis showed level shifts in incidence while preserving the age- and sex-specific shape of the data. The effect was small to moderate across age groups from 50–90 years and negligible at ≤ 45 years, given the reported precision. Absolute shifts were largest at very old ages but remained smaller than the empirical 95% interval widths, supporting the robustness of our qualitative conclusions in the case of plausible global miscalibration. Accordingly, age- and sex-stratified comparisons remain reliable, whereas absolute incidence levels at ≥ 85–90 years should be interpreted with caution (see Results – Sensitivity analysis).

### Literature comparison

Table 2 shows a selected summary of various national and international studies on the incidence of HF and data sources during the period of our estimate.

**Table 2:** Comparison of selected published national and international incidence estimations in the analyzed period from 2009 to 2017.

Author	Country	Year	Incidence	Data	Further information
Zarrinkoub et al. (2013)	Sweden	2010	Age 90 + / women: 42.5/1,000 py (PDE: 44.735/1,000 py [28.957; 61.339]) Age 90 + / men: 51.5/1,000 py (PDE: 87.638/1,000 py [73.286; 101.613])	Administrative health data register including more than 2 million inhabitants	Cross sectional study on individual patient data Age at first HF diagnosis in 2010: 77 ± 13 years, women: 80 ± 12 years; men: 74 ± 13 years
Conrad et al. (2018)	United Kingdom	2002–2014	Standardized by age and sex the incidence of HF for the year 2014 was 3.32/1,000 py Age-standardized incidence decreased overall but increased slightly in the very old (85 + years)	Primary and secondary electronic health records of 4 million individuals from the Clinical Practice Research Datalink (CPRD) Cohort is representative for the UK population in terms of age and sex	Age at initial diagnosis: Men: 74 ± 12.7 years Women: 79.4 ± 11.8 years
Ødegaard et al. (2020)	Norway	2013 2016	3.59/1,000 py (Crude Incidence Rate) 3.44/1,000 py (Crude Incidence Rate)	The Norwegian Drug Prescription Database (NorPD)	39.98/1,000 py for men and 22.81/1,000 py for women in the age group ≥90 years of age (2016)
Störk et al. (2017)	Germany	2011	655/100,000 (≙ 6.55/1,000) persons at risk	Data from the German Health Risk Institute (HRI) from 2009 to 2013: Patients with at least two heart failure-related diagnoses in 2011: ICD-10-GM codes (e.g., I50.0, I50.1) or else: newly diagnosed	Method: Disease-free intervals Increasing incidence with age that was similar for both sexes
Holstiege et al. (2023)	Germany	2013	Cumulative age- and sex-standardized incidence of 10.49 cases per 1,000	Data of the Central Institute for Statutory Health Insurance in Germany (Zi)	Method: Disease-free intervals

### Comparison of incidence estimations

Only limited and inconsistent data on the incidence rate of HF are available in Germany. Holstiege et al. published an incidence estimation based directly on Zi data for the years 2013–2021 [13]. In 2013, the cumulative incidence of HF in Germany was reported to be 10.49 cases per 1,000 persons. Our analysis included the years 2009–2017, and therefore includes an intersection with the year 2013 as reported by the Zi. However, a direct comparison with the data reported by Zi was only possible to a limited extent: as the Zi only reports cumulative incidences and a median age of 76 years for HF, an attempt to standardize our data for direct comparability failed due to the lack of precise information from the report by Holstiege et al. [13]. Therefore, we believe that our approach provides a more detailed and comprehensive estimate of incidence by age group and gender (Table 1, Fig 3).

In 2017, Störk et al. [11] showed that men had a higher risk of developing HF than women in all age groups, with the highest rates observed in the oldest age groups. Estimated incidence rates reported by Störk et al. were consistently higher than our PDE-based estimates for males younger than the 80–84-year age group. Around this age range, the estimates approach each other (Störk et al.: 36.16/1,000 persons at risk; PDE: 35.72/1,000 py). At older ages, our PDE-based estimates were consistently higher. A similar initial pattern was observed in females, with incidence rates estimated by Störk et al. consistently exceeding our PDE-based estimates in the corresponding age groups; however, there was no adjustment observed in higher age groups. Deviations between the estimates may be related to several factors, including differences in the studied populations. Our PDE-based incidence estimation incorporates a larger patient population due to the use of Zi prevalence data [12], but also carries greater uncertainty in higher age groups due to the reliance on Norwegian mortality data [22]. However, the disease-free intervals (DFIs) method for incidence estimation used by Störk et al. and Holstiege et al. can lead to an overestimation of incidence, especially when only short intervals are used. The reason for this is the lack of time to distinguish prevalent from incident cases. As a result, many existing cases are categorized as “new”. The minimum length for an acceptable estimate (<10% overestimation) of the incidence of HF would be 4 years [14]. This overestimation can be avoided by using PDE-based incidence estimation, where no DFIs are required. Furthermore, more precise information on the incidence rates could also be provided by including a significantly longer observation period using the PDE-based incidence estimation. The PDE approach used in this study enables incidence estimation based on publicly available prevalence and mortality data, offering several advantages. For instance, it eliminates the need for longitudinal cohort studies with patient follow-up, thereby saving time and reducing costs [27].

Zarrinkoub et al. [28] estimated the incidence of HF for the Swedish population. The age-adjusted incidence for HF in the year 2010 was lower in all age groups < 90 years in comparison to our incidence estimate using the PDE.

Conrad et al. [29] reported temporal trends for the incidence of HF. Men exhibited a higher incidence rate than women, with an age-standardized incidence rate 1.5 times greater (IRR: 1.52; 95% CI: 1.50–1.54). The difference was particularly pronounced in younger age groups. For example, in the 45–54 years age group, the incidence rate for men was approximately 2.23 times higher than for women (IRR: 2.23; 95% CI: 2.08–2.39). However, in age groups below 55 years, incidence was very low and did not contribute significantly to the overall rate. Meanwhile, an increase in the incidence was observed in the older age groups, starting from 85 years. These findings in both younger and older groups align with our results. However, the study does not provide precise information on incidence trends by specific age groups or gender, limiting the possibility of a comprehensive comparison.

The publication by Ødegaard et al. [22]—from which we took the MRR for the PDE—reported an incidence rate higher in men than in women and increasing with age. However, compared to our incidence estimates, the HF incidence was lower in all age groups. The median age of newly diagnosed HF was 72 ± 14 years, with a median age of 76 years for women and 70 years for men. For diagnosing HF, the authors relied on prescriptions for HF-specific medications linked to detailed ICD-10 codes over a four-year period.

The PDE method has been successfully applied in previous studies to estimate the incidence of various chronic conditions, including diabetes mellitus (types 1 and 2), and Parkinson’s disease [16–19,27]. To gain a more accurate understanding of HF, further research on mortality and incidence surveys in Germany is needed. Such studies could provide a better foundation for assessing future trends in HF by age and gender, potentially leading to the development of targeted screening measures. Additionally, the incidence rates estimated using the PDE could serve as the basis for projecting future HF case numbers and developing prevention programs; this has been previously reported by Tönnies et al. for diabetes mellitus type 2 [30].

### Limitations

The Zi data used included 70 million people with statutory health insurance in Germany. Approximately 12.5% of the German population is privately insured and not included in the Zi data. Statutory insurance enrollees systematically differ from private members in morbidity, service use, and care pathways, implying a credible risk of selection bias [23,24]. However, the direction and magnitude of any such bias are uncertain. If private beneficiaries have lower HF morbidity at older ages, statutory-based estimates could overstate whole-population incidence. Conversely, differential detection/coding or access could lead to underestimation. However, to our knowledge, the private-specific HF data that would be required for direct adjustment are currently unavailable. Another potential limitation is that aggregated rather than individual-level data were analyzed. The included ICD codes cover both the primary diagnosis of HF and other conditions associated with its development. Our analysis was also limited to these ICD codes, preventing us from estimating the incidence for the primary diagnosis separately. This could potentially lead to bias and result in an overestimation of both prevalence and the derived incidence. The Zi data were derived from contract doctors of statutory health insurance. As a result, these diagnoses were not exclusively made by cardiologists, but also by general practitioners. This could affect the diagnostic reliability.

For the estimated age- and sex-specific incidence, we used 2.5% and 97.5% quantiles calculated in 5,000 samples as 95% bootstrap CIs. Due to the uncertainty of the MRR for HF derived from the Norwegian data, the estimation of incidence was subject to increasing uncertainty with age, which is reflected in the widening confidence intervals for higher age groups. Our sensitivity analysis probed only a global multiplicative calibration error of the Norwegian MRRs, and did not assess age- or sex-specific deviations in their shape. General population mortality was treated as fixed, and the same random draws were reused across scenarios to enable paired comparisons. Given the widening empirical 95% intervals at very old ages, inference on absolute level shifts ≥ 85–90 years remains less precise. One possible explanation for this may be the smaller number of patients in older age groups of the Norwegian database, since the CIs were not based on the higher number of prevalence data used for HF (with over 70 million insured persons).

Using the Zi dataset leads to further limitations affecting our estimation. The data is based on billing data from contract physicians of the Associations of Statutory Health Insurance Physicians (“Kassenärztliche Vereinigung”). Therefore, the problem of upcoding cannot be completely excluded [31]. Since the data collection was conducted on an outpatient basis, this limitation is considered likely negligible. Upcoding would be more probable in inpatient data collection due to the economic interests of large healthcare providers [32,33].

A further weakness of the data is that the diagnoses were carried out on an outpatient basis only, without including inpatient diagnoses. This results in a weakness of the method of incidence estimation based on the PDE, as it cannot detect sudden changes between 2009 and 2017. However, evidence from the 2020 care report [34] suggests there were no disruptive changes in heart disease.

## Conclusion

Based on the data from 70 million health-insured individuals in Germany, an IDM and a related PDE were used for the first time to estimate the incidence of HF for men and women ranging from 20 to over 90 years of age between 2009 and 2017. The results showed that regardless of age, men had a higher incidence of HF than women. The highest median incidence was estimated in the age group over 90 years. To test the uncertainty of the estimates, 95% bootstrap CIs were used and showed increasing imprecision in the older age groups. These findings underscore the importance of age- and sex-specific approaches in HF prevention and management strategies, particularly in older populations, for whom the burden of HF is most pronounced.

### Outlook

Incidence estimation is often based on expensive longitudinal cohort studies. Research using DFI-based methods often produces overestimates due to observation intervals being too short, leading to prevalent HF cases being evaluated as incident cases [14]. By using the PDE provided, the problem of DFI and the associated overestimation of HF can be avoided, as can cost-intensive cohort studies. On the basis of existing data on prevalence and mortality, the PDE can be used to get accurate sex- and age-specific estimations of incidence for chronic diseases like HF. As we observed a sharper increase in incidence rates in men from around the age of 60 and in women from the age of 65, this may provide clues as to suitable screening ages. This could help avert the severe consequences of HF at an early stage. Therefore, the PDE method could be used in the future to determine effective screening and monitoring programs by age and gender, helping to minimize the burden of disease and high treatment costs. Our results provide a suitable foundation on which further research can provide more accurate information on appropriate screening ages.

## Supporting information

S1 Text Literature search with MEDLINE. The following search was performed in MEDLINE (PubMed Advanced Search Builder) to identify the study by Ødegaard et al. [22] “Incidence, prevalence, and mortality of heart failure: a nationwide registry study from 2013 to 2016”.
(DOCX)

S1 Fig Regression analysis of the MRR Data used for the PDE. Regression analysis of the published MRR data by Ødegaard et al. [22] for the estimation of German incidence using the PDE. Adjusted R² = 0.9271.
(DOCX)

S1 Table Sensitivity of German incidence estimates to calibration of Norwegian MRRs. **Sensitivity analysis for the scenario MRR + 15%.** Sensitivity analysis of German incidence estimates to calibration of Norwegian mortality rate ratios (MRR + 15% scenario). Age- and sex-specific incidence of heart failure per 1,000 person-years is reported as bootstrap medians with 95% empirical intervals for 5-year age groups.
(DOCX)

S2 Table Sensitivity of German incidence estimates to calibration of Norwegian MRRs. **Sensitivity analysis for the scenario MRR-15%.** Sensitivity analysis of German incidence estimates to calibration of Norwegian mortality rate ratios (MRR-15% scenario). Age- and sex-specific incidence of heart failure per 1,000 person-years is reported as bootstrap medians with 95% empirical intervals for 5-year age groups.
(DOCX)

## Abbreviations

HF: Heart Failure
IDM: Illness-death model
PDE: Partial differential equation
Zi: Central Institute for Statutory Health Insurance (Zentralinstitut für die kassenärztliche Versorgung in der Bundesrepublik Deutschland)
CI: Confidence intervals
HMD: The Human Mortality Database
MRR: Mortality rate ratio
SMR: Standardized mortality ratio
py: person-years
DFI: Disease-free intervals
IRR: Incidence rate ratio
ICD: International Statistical Classification of Diseases and Related Health Problems
