# Supplementary material for: Estimating the incidence of heart failure: Insights from an illness-death model using statutory health insurance data from 70 million people in Germany
Source: PLoS One. 2026 Feb 2;21(2):e0341810. doi: 10.1371/journal.pone.0341810 (PMC12863671; doi:10.1371/journal.pone.0341810)
Supplement: S1 Text — The following search was performed in MEDLINE (PubMed Advanced Search Builder) to identify the study by Ødegaard et al. [22] “Incidence, prevalence, and mortality of heart failure: a nationwide registry study from 2013 to 2016”. (DOCX) [file pone.0341810.s001.docx]

**Literature search with MEDLINE:**

The following search was performed in MEDLINE (PubMed Advanced Search Builder) to identify the study by Ødegaard et al. [22] "Incidence, prevalence, and mortality of heart failure: a nationwide registry study from 2013 to 2016":

Query box keywords:

(((((Incidence[Title/Abstract]) AND (Prevalence[Title/Abstract])) AND (Mortality[Title/Abstract])) AND (Heart Failure[Title/Abstract])) AND (Nationwide[Title/Abstract])) AND (Epidemiology).

Results:

55 results with the selected study by Ødegaard et al. [22] in third position.

*Date and time of search:*

09/09/2024, 8:32 a.m.
