# Supplementary material for: Estimating the incidence of heart failure: Insights from an illness-death model using statutory health insurance data from 70 million people in Germany
Source: PLoS One. 2026 Feb 2;21(2):e0341810. doi: 10.1371/journal.pone.0341810 (PMC12863671; doi:10.1371/journal.pone.0341810)
Supplement: S1 Fig — Regression analysis of the published MRR data by Ødegaard et al. [22] for the estimation of German incidence using the PDE. Adjusted R² = 0.9271. (DOCX) [file pone.0341810.s002.docx]

**Regression analysis of the MRR-Data used for the PDE:**


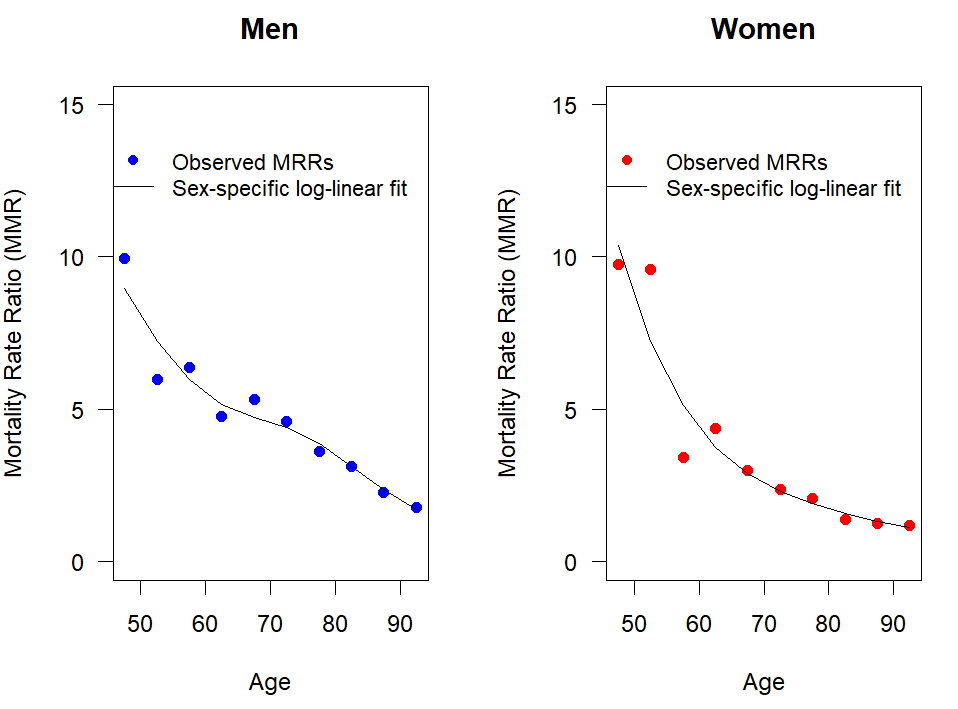
 **S1 Fig**.: Regression analysis of the published MRR data by Ødegaard et al. [22] for the estimation of German incidence using the PDE. Adjusted R² = 0.9271.
