# Supplementary material for: Estimating the incidence of heart failure: Insights from an illness-death model using statutory health insurance data from 70 million people in Germany
Source: PLoS One. 2026 Feb 2;21(2):e0341810. doi: 10.1371/journal.pone.0341810 (PMC12863671; doi:10.1371/journal.pone.0341810)
Supplement: S1 Table — Sensitivity analysis for the scenario MRR + 15%. Sensitivity analysis of German incidence estimates to calibration of Norwegian mortality rate ratios (MRR + 15% scenario). Age- and sex-specific incidence of heart failure per 1,000 person-years is reported as bootstrap medians with 95% empirical intervals for 5-year age groups. (DOCX) [file pone.0341810.s003.docx]

**Sensitivity of German incidence estimates to calibration of Norwegian Mortality rate ratios (MRRs). Sensitivity analysis for the scenario MRR+15%:**

| **Age (years)** | **Males** | **95%-CI** | **Females** | **95%-CI** |
| --- | --- | --- | --- | --- |
| **20 - 24** | 0.04 | [0.04; 0.04] | 0.03 | [0.03; 0.03] |
| **25 - 29** | 0.08 | [0.07; 0.08] | 0.05 | [0.05; 0.05] |
| **30 - 34** | 0.14 | [0.14; 0.14] | 0.09 | [0.09; 0.09] |
| **35 - 39** | 0.27 | [0.27; 0.28] | 0.15 | [0.15; 0.16] |
| **40 - 44** | 0.51 | [0.50; 0.53] | 0.28 | [0.27; 0.28] |
| **45 - 49** | 0.98 | [0.96; 1.00] | 0.51 | [0.50; 0.52] |
| **50 - 54** | 1.85 | [1.81; 1.91] | 0.95 | [0.93; 0.98] |
| **55 - 59** | 3.43 | [3.33; 3.55] | 1.76 | [1.71; 1.83] |
| **60 - 64** | 6.12 | [5.89; 6.38] | 3.20 | [3.08; 3.35] |
| **65 - 69** | 10.36 | [9.86; 10.89] | 5.57 | [5.28; 5.91] |
| **70 - 74** | 16.46 | [15.47; 17.47] | 9.10 | [8.44; 9.83] |
| **75 - 79** | 25.15 | [23.24; 27.09] | 14.26 | [12.81; 15.87] |
| **80 - 84** | 38.46 | [34.78; 42.17] | 22.10 | [18.93; 25.57] |
| **85 - 89** | 60.20 | [53.00; 67.28] | 34.19 | [27.07; 41.72] |
| **90+** | 97.45 | [83.16; 111.19] | 52.14 | [35.83; 68.88] |

**S1 Table:** Sensitivity analysis of German incidence estimates to calibration of Norwegian mortality rate ratios (MRR+15% scenario). Age- and sex-specific incidence of heart failure per 1,000 person-years is reported as bootstrap medians with 95% empirical intervals for 5-year age groups.
