# Supplementary material for: Estimating the incidence of heart failure: Insights from an illness-death model using statutory health insurance data from 70 million people in Germany
Source: PLoS One. 2026 Feb 2;21(2):e0341810. doi: 10.1371/journal.pone.0341810 (PMC12863671; doi:10.1371/journal.pone.0341810)
Supplement: S2 Table — Sensitivity analysis for the scenario MRR-15%. Sensitivity analysis of German incidence estimates to calibration of Norwegian mortality rate ratios (MRR-15% scenario). Age- and sex-specific incidence of heart failure per 1,000 person-years is reported as bootstrap medians with 95% empirical intervals for 5-year age groups. (DOCX) [file pone.0341810.s004.docx]

**Sensitivity of German incidence estimates to calibration of Norwegian Mortality rate ratios. Sensitivity analysis for the scenario MRR-15%:**

| **Age (years)** | **Males** | **95%-CI** | **Females** | **95%-CI** |
| --- | --- | --- | --- | --- |
| **20 - 24** | 0.04 | [0.04; 0.04] | 0.03 | [0.03; 0.03] |
| **25 - 29** | 0.07 | [0.07; 0.08] | 0.05 | [0.05; 0.05] |
| **30 - 34** | 0.14 | [0.14; 0.14] | 0.09 | [0.09; 0.09] |
| **35 - 39** | 0.26 | [0.26; 0.27] | 0.15 | [0.15; 0.15] |
| **40 - 44** | 0.50 | [0.49; 0.51] | 0.27 | [0.27; 0.28] |
| **45 - 49** | 0.95 | [0.93; 0.97] | 0.50 | [0.49; 0.51] |
| **50 - 54** | 1.78 | [1.74; 1.82] | 0.92 | [0.90; 0.95] |
| **55 - 59** | 3.25 | [3.16; 3.34] | 1.70 | [1.66; 1.75] |
| **60 - 64** | 5.73 | [5.53; 5.94] | 3.07 | [2.96; 3.18] |
| **65 - 69** | 9.54 | [9.11; 10.00] | 5.25 | [5.01; 5.54] |
| **70 - 74** | 14.82 | [13.95; 15.74] | 8.39 | [7.85; 9.02] |
| **75 - 79** | 22.04 | [20.29; 23.86] | 12.73 | [11.50; 14.14] |
| **80 - 84** | 32.60 | [29.12; 36.20] | 18.83 | [16.05; 21.97] |
| **85 - 89** | 49.11 | [42.11; 56.20] | 27.11 | [20.71; 34.15] |
| **90+** | 76.16 | [61.93; 90.23] | 36.38 | [21.29; 52.62] |

**S2 Table:** Sensitivity analysis of German incidence estimates to calibration of Norwegian mortality rate ratios (MRR-15% scenario). Age- and sex-specific incidence of heart failure per 1,000 person-years is reported as bootstrap medians with 95% empirical intervals for 5-year age groups.
